# Foreign

**Published:** 1841-03-06

**Authors:** 


### FOREIGN.

*Observations on the Diagnosis and Pathology of Fractures of the Neck of the Femur.* By Robert William Smith, A. M., M. R. I. A.—

[This is a very valuable memoir, well deserving attentive perusal. We regret that we can only find room for the tabular view of the cases (each of which is detailed in the paper and illustrated by a wood-cut,) and the conclusion deduced therefrom by the author.]

INTRACAPSULAR FRACTURES OF THE NECK OF THE FEMUR.

| *No.* | *Name.* | *Age.* | *Shortening.* | *Position of the Foot.* | *Period of Survival after the Receipt of the Injury.* |
|---|---|---|---|---|---|
| 1 | Laurence Maguire - - - | 40 | ½ inch. | Eversion. | 14 days. |
| 2 | William Collins - - - | 36 | ¾ " | " | 17 " |
| 3 | Thomas Maguire - - - | 84 | ½ " | " | 14 " |
| 4 | Dorah Campbell - - - | 75 | 1 " | " | 2 months. |
| 5 | Mary Gill - - - - | 80 | ½ " | " | Not known. |
| 6 | Esther Christie - - - | 60 | 1½ " | " | " |
| 7 | Mary Lamb - - - - | 80 | ¾ " | " | 1 year. |
| 8 | Margaret Bourke - - - | 90 | ½ " | " | 14 days. |
| 9 | Margaret Myler - - - | 78 | ¼ " | " | 2 months. |
| 10 | A Female - - - - | 65 | 1 " | " | Not known. |
| 11 | Patrick Doolan - - - | 60 | 2 " | " | 7 years. |
| 12 | Michael Curry - - - | 40 | 1¼ " | " | 1 month. |
| 13 | Matthew Reilly - - - | 46 | ½ " | " | 4 months. |
| 14 | A Female - - - - | 55 | 1 " | " | Not known. |
| 15 | Sarah Ashton - - - | 65 | 1¼ " | " | 9 years. |
| 16 | Elizabeth Casey - - - | 50 | ¾ " | Inversion. | Several years. |
| 17 | Robert Robinson - - - | 50 | 2 " | Eversion. | Not known. |
| 18 | Ellen Walker - - - | 70 | ½ " | " | 7 days. |
| 19 | Laurence Reilly - - - | 56 | 2 " | " | Several years. |
| 20 | Joseph Seaton - - - | 90 | 1¼ " | " | 7 years. |
| 21 | A Female - - - - | 65 | 2½ " | " | Several years. |
| 22 | Thomas Connolly - - - | 50 | ¾ " | " | 10 days. |
| 23 | Bridget Missett - - - | 72 | 1 " | " | 10 weeks. |
| | EXTRACAPSULAR FRACTURE OF THE NECK OF THE FEMUR. | | | | |
| 24 | Patrick Murphy - - - | 80 | 2 inch. | Inversion. | 14 days. |
| 25 | Alicia Harris - - - | 70 | 1½ " | Eversion. | 5 " |
| 26 | James Stanford - - - | 67 | 2 " | " | 8 " |
| 27 | A. B., a man - - - | 50 | 2 " | " | 14 " |
| 28 | Mary Kelly - - - - | 56 | 1¼ " | " | 11 " |
| 29 | Ellen Bryan - - - - | 65 | 1½ " | " | 5 weeks. |
| 30 | Patrick Grant - - - | 70 | 1½ " | " | 5 days. |
| 31 | Margaret Connolly - - | 89 | 1½ " | " | 12 " |
| 32 | Thomas Murphy - - - | 41 | Not known. | " | A few weeks. |
| | IMPACTED FRACTURES OF THE NECK OF THE FEMUR, EXTERNAL TO THE CAPSULE. | | | | |
| 33 | John Summers - - - | 74 | 1½ inch. | Eversion. | 2 months. |
| 34 | Mary M'Kenna - - - | 52 | ¾ " | " | 4 days. |
| 35 | Catharine Egan - - - | 60 | 1¼ " | Inversion. | 1 month. |
| 36 | Sarah Denny - - - | 70 | 1 " | Eversion. | 1 " |
| 37 | Alicia Sherlock - - - | 64 | ½ " | " | 15 weeks. |
| 38 | James Power - - - | 54 | 1½ " | " | 5 months. |
| 39 | A. B., a female - - - | 80 | Not known. | | Not known. |
| 40 | Not known - - - - | 00 | Not known. | | Not known. |
| 41 | Bryan Dunn - - - - | 60 | ¼ inch. | " | 13 days. |
| | IMPACTED FRACTURE OF THE NECK OF THE FEMUER, INTERNAL TO THE CAPSULE. | | | | |
| 42 | Owen Curran - - - | 70 | ½ inch. | Eversion. | 1 year and 10 mo. |

From what has been stated in the preceding pages, and from the evidence afforded by the *post-mortem* examination of fifty specimens of fractures of the neck of the femur, forty-two of which have been detailed, I think I am justified in deducing the following conclusions:

1. A slight degree of shortening, removable by the extension of the limb, indicates a fracture within the capsular ligament. 2. The degree of shortening, where the fracture is within the capsular ligament, varies from a quarter of an inch to one inch, or one inch and a half. 3. The degree of shortening, when the fracture is within the capsule, varies chiefly according to the extent of laceration of the fibro-synovial folds which invest the neck of the femur. 4. In some cases of intracapsular fracture the injury is not immediately followed by shortening of the limb. 5. This absence of shortening is generally owing to the integrity of the fibro-synovial folds. 6. In such cases the retraction of the limb may occur suddenly, many weeks after the receipt of the injury. 7. This sudden retraction of the limb, which indicates a fracture within the capsule, is, in general, to be ascribed to the accidental laceration of the fibro-synovial folds. 8. The degree of shortening, when the fracture is external to the capsule and not impacted, varies from one inch or one inch and a half, to two inches or two inches and a half. 9. When a great degree of shortening occurs immediately after the receipt of the injury, we usually find a comminuted fracture external to the capsule. 10. The extracapsular fracture is generally accompanied by fracture with displacement of one or both trochanters. 11. The extracapsular *impacted* fracture is generally accompanied by fracture without displacement of one or both trochanters. 12. In such cases the fracture of the processes unites more readily than that of the cervix. 13. The degree of shortening, when the fracture is impacted, varies from a quarter of an inch to one inch and a half. 14. The exuberant growths of bone met with in these cases have been by many erroneously considered to be merely for the purpose of supporting the acetabulum and the neck of the femur. 15. The difficulty of ascertaining crepitus, and of restoring the limb to its natural length, are the chief diagnostic signs of the impacted fracture. 16. The position of the foot is as much influenced by the obliquity of the fracture and the relative position of the fragments, as by the action of the muscles. 17. Inversion of the foot may occur in the intracapsular, extracapsular, or impacted fracture of the neck of the femur. 18. When in the intracapsular fracture the lower fragment is placed in front of the upper, the foot is usually inverted. 19. When in the extracapsular fracture with impaction, the superior is driven into the inferior fragment, so as to leave the greater portion of the latter in front of the former, the foot is generally inverted. 20. In cases of comminuted extracapsular fracture without impaction, but with separation and displacement of the trochanters, the foot may be turned either inwards or outwards, and will generally remain in whatsoever position it has been accidentally placed. 21. The consolidation by bone of the intracapsular fracture is most likely to occur, when the fracture is also impacted. 22. Severe contusion of the hip-joint, causing paralysis of the muscles which surround the articulation, is liable to be confounded with fracture of the neck of the femur. 23. The presence of chronic rheumatic arthritis may not only lead us to suppose that a fracture exists when the bone is entire, but also when there is no doubt as to the existence of fracture, may render diagnosis difficult as to the seat of the injury with respect to the capsule. 24. Severe contusion of the hip-joint, previously the seat of chronic rheumatic arthritis, and the impacted fracture of the neck of the femur, are the two cases most liable to be confounded with each other. 25. Each particular symptom of fracture of the neck of the femur, separately considered, must be looked upon as equivocal: the union of all can alone lead to correct diagnosis.—*Dublin Journal of Medical Science.*

---

*Angina Pectoris, terminating fatally by Rupture of the right Coronary Artery.* By Dr. A. Gallardi.—A shoemaker, aged about 40, was received into the Milan Hospital, May 21, 1834. He was of good constitution, well formed, of rather nervous temperament, and somewhat addicted to strong drinks. He said that for some months past he had at times, while at work, felt some difficulty of breathing, with a loss of power in the left arm, and subsequent slight lipothymia. Twice in the last month, on going out of his cottage into the open air, he had felt severe pain in the heart, extending to the left scapula and arm, and accompanied by a momentary reeling and slight obscuring of the senses. These symptoms had occurred with unusual severity two days before his admission, after drinking some spirits, and were followed by a death-like syncope; from which, however, he was quickly and completely restored by a large bleeding.

On the day of his admission the patient was found suffering from severe dyspnœa, of a character which led the author at once to suspect a disease of the heart, although it was combined with a number of anomalous symptoms that could only be referred to hypochondriasis. On percussion, both sides of the chest had a natural resonance; but in the median line and the adjacent part of the left side, there was dulness over even a greater extent than in most cases of hypertrophy with hydropericardium. The movements of the heart were scarcely perceptible to the hand placed on the cardiac region, but posteriorly they were even more sensible than natural. The pulse was in no degree intermitting, but small, sometimes very frequent, at others quick, and very hard. The patient was bled to twelve ounces and had other antiphlogistic remedies, and on the following day was much better. On the 23d, however, all his symptoms were worse than ever; he had excessive dyspnœa, severe pain in the heart, constant dry cough, and frequent faintings. The heart could be only very obscurely felt in front, but close by the vertebral column its action was more than ever distinct; the pulse was still very small and hard, and the left hand and forearm were œdematous. The patient was repeatedly bled and subjected to severe antiphlogistic treatment, but all his symptoms continued or grew worse; the œdema spread gradually over the whole body, and on the 31st he died suddenly in a fit of syncope.

The body was examined thirty hours after death. The contents of the skull presented nothing unusual. The pleural cavities contained a large quantity of serous fluid; the lungs were both œdematous, and their lower lobes were much compressed both by the fluid and the immensely dilated pericardium. This sac was evidently distended by some dense consistent fluid containing small grumous masses, and pressing the heart unnaturally far backwards. On cutting it open along its anterior wall, a large quantity of grumous blood and serum flowed from the pericardium; and "at the aperture thus made there soon appeared a body whose surface was of a whitish colour, beset with innumerable uniform oblong appendices, which might remind one of the surface of the villous heart, as described by pathological anatomists. This pseudo-organism, which was of a conical form, was fixed on the heart, and adhered by its base to the base of the ventricle and the right auricle: it was as large as the heart itself. On washing the parts, it was found that the base of this tumour adhered to the parts just mentioned by false membranes, and that it had obtained by them an adhesion to the right coronary artery, in which there was a laceration about two lines in length with elevated edges;" through which the blood had been poured into the pericardium, and, becoming organized, had been developed into this remarkable tumour. At the left side the adhesions by which the tumour had before been attached to the heart had been broken down, and thus the exit of the additional quantity of blood which had been found in the pericardium had been permitted. The heart itself, and its valves, were perfectly healthy, but the aorta and its great branches presented signs of arteriasis, and there was a cartilaginous and osseous elevation close by the origins of the coronary arteries: the pulmonary artery and its branches were similarly diseased. The contents of the abdomen were generally healthy.

To this case, which we believe is unique, the author has added very lengthened observations, of which, however, those only are sufficiently important which refer to the probability that the rupture of the coronary artery and the first effusion of blood took place when the patient was seized with the very severe symptoms two days before his admission, and that the second and fatal effusion occurred at the instant of the fainting fit, in which the patient's life was terminated. It is to be regretted that the general condition of the coronary vessels is not mentioned.—*British and For. Med. Rev., from Annali Universali di Medicina.*

---

*On Tubercles of the Larynx and Fauces.* By Dr. Popken, of Jever.—The disease to which I have given this name, at the risk of its being objected that it does not consist of true tubercles, but only of diseased mucous follicles, has often presented itself to me since my attention was first drawn to it, and exhibits so much that is peculiar in its phenomena and course that I cannot refer it precisely to any of the generally known chronic diseases of the larynx. I have never seen it before puberty, nor in advanced age, but always in young subjects, and chiefly in males; neither could I ever trace any connexion between this and any constitutional chronic diseases, as syphilis or scrofula. I have so rarely found it coincident with other local affections, and especially with those of the lungs, that, in a doubtful diagnosis, I rather regard this disease in the throat as a sign that there is *not* a vomica in the lungs.

In general the patient, in whom this peculiar alteration of the mucous membrane lining the fauces and larynx exists, begins, without any evident cause, to cough and hawk very frequently: but he complains of no pain in the affected part, and is usually in perfectly good general health, so that the whole disorder appears like an obstinate cold, and is annoying only from the incessant irritation that excites the hawking. The disease may thus continue apparently without alteration for weeks or months, till at last (and usually in the morning after drinking) the ejection of one or more small masses of an elastic matter, just like chalcedony in colour and semitransparency, attracts the patient's attention, and, though followed by temporary relief, makes him anxious.

If the fauces be examined at this period of the disease, one finds the whole of their posterior wall thickly beset with small, round, bright red, semitransparent bodies, varying in size from that of a pin's head to that of a pea, and just like the granulations which Laennec and Louis describe as the commencement of tubercles. On some of these granulations one sees adhering firmly to them the opaline mucous globules already mentioned, and just similar in size and form to the bodies beneath them. This granulated condition of the mucous membrane, which, as far as I know, has not been before remarked by any one, extends down as far as the eye can reach, and is always a certain sign of the existence of similar tubercles in the mucous membrane of the larynx, and of threatening laryngeal phthisis; for, although the latter disease does not necessarily, and still less rapidly follow, yet instances of its occurrence, as a signal of the milder affection just described, are not wanting.

This fatal result of the disease, however, may, the author asserts, be always averted by early and judicious treatment. The means which he has employed with the best success are a nutritious, but not stimulating diet, constant warmth round the throat, and the administration of aromatic and tonic medicines, such as ammoniacum and myrrh, or myrrh, extract of bark, and sulphate of iron.

[The disease described by the author must be familiar to every practitioner, but we are not aware that the anatomical condition on which it depends has been before pointed out, though it is distantly alluded to by Henle (Ueber Schleim-und-Eiterbildung, in Hufeland's Journal,) who found the expectorated substance to consist of morbidly altered epithelium cells. The name which the author has given it is most objectionable, expressing only what the disease is *not;* it is an affection of the mucous membrane, exactly analogous to acne of the skin, and its name should express, if possible, that it has its origin in disease of the mucous-glands.]—*Ibid., from Casper's Wochenschrift.*

This is nothing but the common pharyngitis, which has of late years been so prevalent in this country. The follicles are inflamed and prominent, but not independently of the intervening mucous membrane. The larynx may be affected from the extension of the disease from the pharynx, but the large majority of cases do not end in laryngeal phthisis. Occasionally, however, this result takes place. The term tubercle is a ridiculous one—the treatment is a correct one.

---

*Dropsy after Scarlatina, unaccompanied with Albuminous Urine.* By Dr. Philipp, of Berlin.—During the spring of the year 1840, scarlet fever was very prevalent in Berlin, especially in the northern and eastern parts of the city. A hundred cases of the disease came under Dr. Philipp's notice, who found that it resembled the late London epidemic, in being invariably followed by dropsy. Some cases of the fever, indeed, terminated fatally from head affection; in other, and more numerous instances, the patients died from putrid sore throat; but whenever that was not the case, dropsical symptoms, more or less severe, occurred from the twelfth day up to the fourth or fifth week after desquamation had commenced. In some children, too, dropsy came on without being preceded by any symptoms whatever of illness.

There were two points in which the Berlin epidemic differed from that which prevailed in London; one was, that, instead of being a fatal disease, not one child died; the other, that, although the author tested the urine in sixty cases, he never detected the presence of albumen by heat alone, and it was only in a few cases that nitric acid gave signs of its presence.

He states, as in some measure accounting for this, that albuminous urine in dropsy is very rare in Berlin; for, during two years in which he has been physician to the poor in one district of the city, although he has had 150 new cases in a month, he has met with but two instances of Bright's kidney.—*Ibid., from Casper's Wochenschrift.*

---

*Case of immense Cæcal Fistula in the Lumbar Region.* By Dr. Meding, of Meissen.—A strong man, aged thirty-six, suffered for some days from griping sensations in the abdomen; on the sixth day I found him with the most complete symptoms of a severe psoitis of the right side, which I believed was the result of either rheumatism from cold, or of a sprain from over-exertion. General and local abstractions of blood, several times repeated, mitigated the pain, but produced no essential change in the state of the patient; a constant restlessness prevented him from sleeping; his pulse was accelerated, but full; his breathing anxious; his tongue moist and furred; he was thirsty and had lost his appetite; his skin was alternately cold and hot; his bowels very loose; his urine depositing an abundant red sediment.

The severe pains in the back from the right lumbar region to the shoulder, and forwards to the testis and the thigh, as well as the great tenderness strictly limited to the right iliac and inguinal region, suggested an affection of the cæcum. But although as the disease went on, there was every reason to believe that effusion or suppuration must have taken place, yet no part could be found at which the matter might be evacuated. Only on the back, the common belly of the sacro-lumbalis and longissimus dorsi appeared somewhat prominent, though without any alteration in the colour or elasticity of the skin. At this part, however, the author determined to make an exploratory incision, and on the 24th day of the disease he cut along the outer edge of the common belly of the muscles just mentioned for four inches upwards from the crista ilii, through the skin and a thick layer of fat down to the external oblique. All these tissues were perfectly healthy; but on introducing a lancet an inch deep into the fibres of the abdominal muscles, a little pure pus gushed out with the blood, and was soon followed by a stream of blackish-gray ichor of a most fetid macerated bone-like smell. For several days the discharge continued to be very profuse, and the patient became extremely weak. Every day there came away with the acrid irritating ichor masses of cellular tissue, partly from the interstices of the outer muscles of the back, and partly from the fat and cellular substance surrounding the kidney and psoas muscle. The finger could now be easily passed over the transverse processes to the anterior surface of the bodies of the lumbar vertebræ, and the psoas and iliacus muscles could be distinctly felt quite cleared of cellular tissue. Such examinations always excited the severest cramps of the chest, and twitchings of the right lower extremity. With a long probe it was found that on the inner side the whole lumbar region of the abdomen behind the peritoneum from the kidney into the cavity of the pelvis, and on the outer side the interspaces of the dorsal muscles from the inferior angle of the right scapula to the middle of the outer surface of the ilium, formed one cavity, the source of the putrid fluid.

For six weeks after the operation the condition of the patient seemed hopeless; he became weaker and more emaciated, had severe spasms of the pharynx and rectum, and was in a state of utter prostration. At the end of this time, however, and quite unexpectedly, the sloughing away of the cellular tissue gradually ceased, the wound assumed a more healthy aspect, the matter discharged became thicker and more purulent, acquired a stercorous odour, and soon after was found to contain small portions of fæces. At the same time the general condition of the patient improved.

The whole of the cavity above described was filled twice a day with dry charpie pushed into it as far as possible with a long and thick piece of whalebone. On passing the latter as a probe forwards and downwards over the crista ilii, one came in the neighbourhood of the fifth lumbar transverse process, immediately at the outer edge of the quadratus lumborum, to an aperture as big as a large quill, through which the whalebone passed to a depth of six or eight inches, till it was stopped by a soft yielding substance. There could be no doubt that this hole led into the cæcum; and it was found that by closing it accurately with charpie, and keeping the patient on his left side, with a careful diet and open bowels, the evacuation of fecal matter through the wound was entirely prevented. With some unimportant interruptions, the gradual closure of the cavity went on steadily after the eighth week from the operation. The quantity of charpie necessary to fill it up (which had at first been very considerable) decreased with the diminution of the discharge; the thigh which had been fixed in the flexed posture became more and more moveable; and all the functions of the body were gradually restored to their natural state. In rather more than ten months from the day on which the cavity was opened, the external wound was completely healed; no relapse took place; the muscles which had been implicated in the disease gradually gained power, and in rather less than two years from the commencement of his malady, the patient was restored to his former robust health.—*Ibid.*, *from Von Ammon's Monatsschrift für Medicin, u. a.*

---

*Apoplexy, with Hemiplegia, in consequence of Fright.* By Dr. Ritter.—The only peculiarity of this case is its cause. A robust and rather plethoric woman, thirty-eight years old, was in perfect health and speaking to a neighbour, when her servant girl frightened her by brandishing a bright spiral wire over her head, so as to make it look as if a snake were falling on her. In her fright the woman suddenly fell down as in an apoplectic fit, and remained for some time nearly unconscious. When examined she complained of a noise and beating in the left side of her head, deafness of the left ear, and of blindness and loss of taste on the same side. She could not move any part of the left side of the body, and in every respect resembled a patient suffering from hemiplegia in consequence of sanguineous apoplexy. By active antiphlogistic treatment, and various other measures, she was gradually restored from this state in about three months.—*Ibid.*, *from Medicinische Zeitung. September* 9, 1840.

---

*Observations on the Therapeutic Efficacy of Ammoniuret of Copper in Chorea.* By Dr. Fedele di Fiore.—Two cases are related of genuine chorea to prove the value of this once esteemed remedy, which in Italy passes by the name of "the specific of Stissero." In each case nearly all the usual and most active methods of treatment had been adopted before this was resorted to; bleedings, leeches along the spine, anthelmintics, purgatives, cold baths, and various antispasmodics had all signally failed to produce benefit, when the ammoniuret of copper was commenced. In doses beginning at one-eighth, and gradually increased to half a grain twice a day; the latter effected a gradual but perfect cure; in one case within two months, in the other in one month.—*Ibid.*, *from Annali Clinici dell' Ospedale degl' Incurabili. Ottobre*, 1839.

---

*On Itch and its Treatment.* By Dr. de la Harpe, Chief Physician of the Hospital of Lausanne.—The author gives the following formula for an ointment which he has employed in upwards of 400 patients. It does not appear to irritate the skin, and is said to be better than the liniment of Valentin or the alkaline sulphur ointment of Alibert. The mean duration of treatment was eighteen days in 1836, fifteen in 1837, eleven in 1838, and ten in 1839. The formula is as follows:

Flowers of sulphur, 16 parts; sulphate of zinc, 2; powder of white hellebore, 4; soft soap, 31; lard, 62.

*Ibid.*, *from Gazette Médicale.*

